# Mutual-Aid Mobile App for Emergency Care: Feasibility Study

**DOI:** 10.2196/15494

**Published:** 2020-03-19

**Authors:** Shuo-Chen Chien, Md Mohaimenul Islam, Chen-An Yeh, Po-Han Chien, Chun You Chen, Yen-Po Chin, Ming-Chin Lin

**Affiliations:** 1 Graduate Institute of Biomedical Informatics College of Medical Science and Technology Taipei Medical University Taipei Taiwan; 2 School of Health Care Administration Taipei Medical University Taipei Taiwan; 3 Business Administration National Taiwan University Taipei Taiwan

**Keywords:** technology acceptance model, cardiopulmonary resuscitation, mobile app, emergency care

## Abstract

**Background:**

Improving the quality of patient care through the use of mobile devices is one of the hot topics in the health care field. In unwanted situations like an accident, ambulances and rescuers often require a certain amount of time to arrive at the scene. Providing immediate cardiopulmonary resuscitation (CPR) to patients might improve survival.

**Objective:**

The primary objective of this study was to evaluate the feasibility of an emergency and mutual-aid app model in Taiwan and to provide a reference for government policy.

**Methods:**

A structured questionnaire was developed as a research tool. All questionnaires were designed according to the technology acceptance model, and a Likert scale was used to measure the degree of agreement or disagreement. Moreover, in-depth interviews were conducted with six experts from medical, legal, and mobile app departments. Each expert was interviewed once to discuss feasible countermeasures and suggestions. Statistical Package for the Social Sciences (SPSS version 19; IBM Corp, Armonk, New York) was used to perform all statistical analyses, including descriptive statistics, independent sample *t*-tests, variance analysis, and Pearson correlation analysis.

**Results:**

We conducted this study between October 20, 2017, and November 10, 2017, at the Taipei Medical University Hospital. Questionnaires were distributed to medical personnel, visiting guests, family members, and volunteers. A total of 113 valid questionnaires were finally obtained after the exclusion of incomplete questionnaires. Cronbach α values for self-efficacy (perceived ease of use), use attitude (perceived usefulness), and use willingness and frequency were above .85, meeting the criterion of greater than .70. We observed that the reliability of each subquestion was acceptable and the values for use attitude (perceive usefulness) and use willingness and frequency were more than .90.

**Conclusions:**

The findings suggest that perceived ease of use and perceived usefulness of the app model affect use willingness. However, perceived usefulness had an intermediary influence on use willingness. Experts in law, medical, and technology fields consider that an emergency and mutual-aid model can be implemented in Taiwan. Along with the development of an emergency and mutual-aid app model, we recommend an increase in the number of automated external defibrillators per region and promotion of correct knowledge about CPR in order to decrease morbidity and mortality.

## Introduction

Internet technology has been gaining momentum in all areas, including health care, and it is showing no signs of slowing down anytime soon. Medical organizations have always been trying to improve staff productivity, frictionless communication, and patient satisfaction [1-4]. However, the quality of human lifestyle has notably increased with improvements in science and technology [3,5,6]. Identifying a sophisticated approach to improve work efficiency and health care quality is still a very important issue [3,4]. Recently, with provision of free and faster consultation services to the public, the Taiwanese government is continuously promoting e-service policies. Owing to the prevalence of the internet and broadband technologies, many emerging online services have already been created [7-9]. Several features, including voice mail, email mailbox, online news groups, online banking, online book purchase, online trading, travel itinerary, online learning, and online instant messaging, have gained popularity [10,11]. According to the Foreseeing Innovative New Digiservices-2014 Taiwan Consumer Mobile Device and App Use Behavior Research Survey Report, it is estimated that the number of individuals aged 12 years or older using mobile devices, such as smartphones and tablets, has reached approximately 14.32 million. The penetration rate of smartphones is about 65.4%, and it will reach 79.6% in 2020 [12,13]. The use of mobile devices has spread among not only younger individuals but also middle-aged individuals, and the survey found that the penetration rate among middle-aged individuals has increased [14]. In addition, it is estimated that the number of users is 16.04 million, and about three out of every four people use mobile devices [14].

Owing to long-term traffic congestion in Jerusalem, ambulances are often associated with a delay in the golden rescue time of patients; therefore, Eli Beer, the founder of United Hatzalah, created a rescue team along with friends through mobile satellite positioning systems [15-18]. Ambulance motorcycles are equipped with various first-aid devices. In an emergency rescue situation, the five volunteers closest to the patient are notified of the location. They can receive relevant information from the mobile phone to rescue and prioritize patients on the scene before the arrival of the ambulance to improve the patient survival rate [19-22]. There are a number of such difficult-to-use apps globally that have already received attention, such as PulsePoint in the United States. In fact, these kinds of emergency and mutual-assist apps are useful to professionals who have received first-aid training and are willing to assist strangers to receive first aid at fire stations. With regard to the satellite positioning system in the smartphone, a message is sent to emergency personnel near the patient’s location and volunteers go to the rescue site according to satellite positioning [23]. This voluntary emergency ambulance network relies on extensive personnel support. The high-tech network system can shorten the time for ambulance personnel to arrive at the patient’s location, strive for appropriate time of first aid, and successfully reduce many regrets that can be avoided [24].

The First AED app, which was jointly developed by the Ministry of Health and Welfare and the Taiwan Emergency Medical Association, was officially launched at the end of 2015. It is downloaded and installed on mobile devices to obtain the locations of automated external defibrillators (AEDs) and first-aid materials and obtain other related information. The app includes one-touch dial 119, cardiopulmonary resuscitation (CPR) plus AED teaching, AED search (quickly locate the nearest AED), information on laws, and frequently asked questions. Similar to PulsePoint, an emergency and mutual-aid app could improve the survival rate of patients; however, there is no such app available in Taiwan.

Therefore, the purpose of this study was to understand the willingness and feasibility of implementation of an emergency and mutual-aid app in Taiwan through questionnaires. Expert interviews were also conducted to understand the legal, medical, technical, and other aspects of the feasibility. The findings of this study could be used as a reference for future national policy.

## Methods

### Overview

This study was approved by the Taipei Medical University research ethics board. Participant consent was obtained, and the information of individuals was deidentified. The Institutional Review Board approval number is N2017403068.

### Technology Acceptance Model

**Figure 1:**
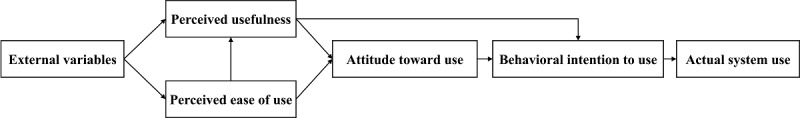
The framework of the technology acceptance model.

The technology acceptance model (TAM) is a set of theories developed by Davis et al [25] in 1989 to explain the determinants of information technology acceptance, especially for technology use behavior. This is based on the theory of rational action, which is widely used in the prediction and interpretation of the acceptance behavior of personal information systems. Davis et al [25] demonstrated that attitude is a very important factor influencing user behavior, and attitude is mainly influenced by the two variables perceived usefulness and perceived ease of use. Perceptual ease of use positively affects perceived usefulness, and perceived ease of use and perceived usefulness affect the attitude toward use, which, in turn, affects behavioral intent to use and the use of information systems (actual systems) (Figure 1).

**Figure 2:**
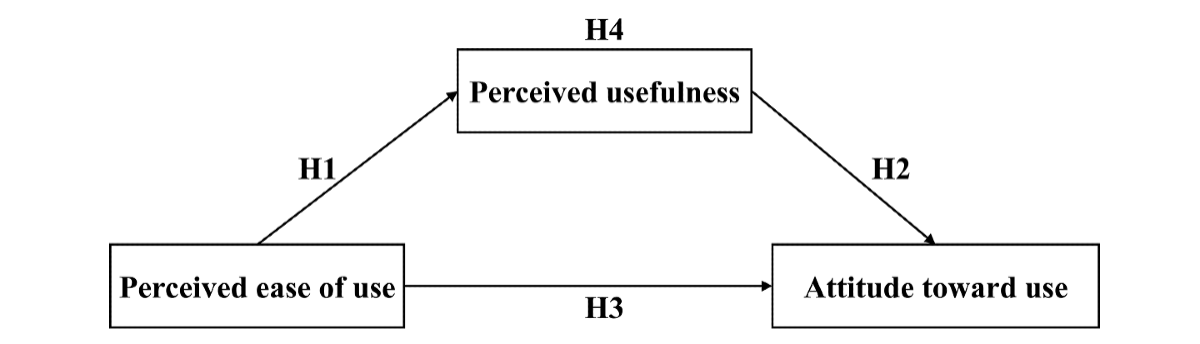
Proposed research model. H1: perceived ease of use and perceived usefulness are significantly positively correlated; H2: perceived usefulness is significantly positively correlated with use willingness and frequency; H3: perceived ease of use is significantly positively correlated with use willingness and frequency; H4: perceived usefulness has a mediating effect on perceived ease of use and use willingness.

According to the TAM in literature verification, we assessed the feasibility factors for the impact of an emergency and mutual-aid app model in Taiwan with self-efficacy (perceived ease of use), use attitude (perceived usefulness), and use willingness and frequency (Figure 2). As a basis for subsequent verification questionnaires and statistical analysis and to verify the research structure and hypothesis, this study proposed the following four hypotheses: (1) perceived ease of use and perceived usefulness are significantly positively correlated; (2) perceived usefulness is significantly positively correlated with use willingness and frequency; (3) perceived ease of use is significantly positively correlated with use willingness and frequency; and (4) perceived usefulness has a mediating effect on perceived ease of use and use willingness.

### Questionnaire Design

This was a cross-sectional study. In addition to individual qualitative interviews, a structured questionnaire was used as a research tool (Multimedia Appendix 1). The questionnaire adopted the TAM as a research method. The questionnaire was developed by reviewing extensive literature and expert suggestions to achieve high content validity. Before implementation of the formal questionnaire, 30 pretest questionnaires were sent to subjects from among teachers and students in the laboratory of Taipei Medical University. Cronbach α values above .70 were considered reliable. The questionnaire had four parts. The first part involved the basic information of the subject. The second involved self-efficacy evaluation for an emergency and mutual-aid app model (perceived ease of use). The third part involved a self-use attitude evaluation for an emergency and mutual-aid app model (perceived usefulness). The fourth part involved a self-evaluation for the willingness and frequency of use of an emergency app model. The total score of a Likert scale (strongly disagree, 1; disagree, 2; neutral, 3; agree, 4; and strongly agree, 5) was used to assess the degree of agreement or disagreement.

### Data Collection

The study was conducted in a university teaching hospital at Taipei Medical University between October 20, 2017, and November 10, 2017. The questionnaires were distributed in a convenient sampling manner to medical personnel, visiting guests, family members, and volunteers. A total of 113 valid questionnaires were collected after excluding incomplete questionnaires.

### Conduction of In-Depth Interviews

To understand the feasibility, advantages, and disadvantages of an emergency and mutual-aid app on mobile phones in Taiwan, individual qualitative in-depth interviews were conducted with six experts from different fields, including medical, legal, and mobile app development fields, according to the results of the self-evaluation questionnaires. It was hoped that findings from a multifaceted perspective would help in the discussion of the feasibility and possible benefits of such an emergency and mutual-aid app at the technical development and legal levels. Feasible countermeasures and suggestions will be discussed in the future.

### Statistical Analysis

Statistical Package for the Social Sciences (SPSS version 19; IBM Corp, Armonk, New York) and Analysis of a Moment Structures (AMOS version 24; IBM Corp) were used to perform all statistical analyses. SPSS was used for descriptive statistics, independent sample *t* tests, variance analysis, and Pearson correlation analysis, and AMOS was used for structural equations and analysis verification.

## Results

### Basic Information

The basic information of the respondents included gender, age, highest education level, and occupation, as well as perceived ease of use, perceived usefulness, and use willingness (Table 1). We distributed questionnaires to 120 participants. A total of 113 respondents completed all questionnaires (63 [55.8%] male and 50 [44.2%] female respondents). Moreover, of the 113 respondents, 40 (35.4%) were aged 20-39 years, 40 (35.4%) were aged 40-59 years, and 33 (29.2%) were aged 60 years or older. Regarding the highest education level, of the 113 respondents, 8 (7.1%) had junior high school or below education, 20 (17.7%) had high school education, 68 (60.2%) had a bachelor’s degree, and 17 (15.0%) had a master’s degree or above.

**Table 1:** Characteristics of the participants (N=113).

Characteristic		Participants, n (%)	
**Gender**			
	Male		63 (55.8)
	Female		50 (44.2)
**Age (years)**			
	20-39		40 (35.4)
	40-59		40 (35.4)
	60 or older		33 (29.2)
**Highest education level**			
	Junior high school or below		8 (7.1)
	Senior high school		20 (17.7)
	Bachelor’s degree		68 (60.2)
	Master’s degree or above		17 (15.0)
**Occupation**			
	Student		1 (0.9)
	Military/public/religious industry		3 (2.7)
	Service industry		14 (12.4)
	Finance industry		2 (1.8)
	Information/technology industry		5 (4.4)
	Communication/advertising/design industry		2 (1.8)
	Art industry		0 (0.0)
	Free industry		9 (8.0)
	Medical care industry		28 (24.8)
	Agriculture/forestry/fishery/animal husbandry industry		1 (0.9)
	Family management/retirement industry		20 (17.7)
	Others		28 (24.8)

Regarding occupation, of the 113 respondents, 1 (0.9%) was a student, 3 (2.7%) were from the military/public/religious industry, 14 (12.4%) were from the service industry, 2 (1.8%) were from the finance industry, 5 (4.4%) were from the information/technology industry, 2 (1.8%) were from the communication/advertising/design industry, 9 (8%) were from the free industry, 28 (24.8%) were from the medical care industry, 1 (0.9%) was from the agriculture /forestry/fishery/animal husbandry industry, 20 (17.7%) were from the family management/retirement industry, and 28 (24.8%) were from other industries.

### Reliability and Correlation Analysis

Cronbach α values for self-efficacy (perceived ease of use), use attitude (perceived usefulness), and use willingness and frequency were above .85, meeting the criterion of greater than .70 (Table 2). When the subquestions were deleted, the Cronbach α values were not significantly larger than the original Cronbach α values (*P*=.54). Therefore, the reliability of each subquestion was acceptable, and the values for use attitude (perceived usefulness) and use willingness and frequency were above .90. These findings indicate that the reliability of the questionnaire content was satisfactory.

Table 3 shows the correlation analysis among the research variables. There were significant correlations between gender and whether medical staff (*r*=0.27;*P*=.003), age and highest education (*r*=−0.45;*P*<001), age and whether medical staff (*r*=−0.26;*P*=005), and highest education and whether medical staff (*r*=0.39;*P*<.001). There were no differences among the respondents’ basic information, such as gender, age, highest education, and whether medical staff. However, there were strong positive correlations between perceived ease of use and perceived usefulness (*r*=0.75;*P*<.001), perceived ease of use and use willingness (*r*=0.76;*P*<.001), and perceived usefulness and use willingness (*r*=0.88;*P*<.001).

**Table 2:** Reliability, mean, and variance analysis.

Variable and measurement project		Mean (SD)	Cronbach α value after the project is deleted	Cronbach α
**Perceived ease of use**		**18.94 (3.98)**		**.85**
	A1	3.49 (1.21)	.86	
	A2	3.85 (0.95)	.80	
	A3	3.96 (0.86)	.81	
	A4	3.90 (0.91)	.82	
	A5	3.74 (1.02)	.81	
**Perceived usefulness**		**20.06 (4.37)**		**.94**
	B1	4.11 (0.88)	.92	
	B2	4.01 (0.98)	.92	
	B3	4.04 (0.93)	.93	
	B4	3.91 (1.04)	.94	
	B5	4.00 (0.98)	.92	
**Use willingness**		**19.71 (4.24)**		**.93**
	C1	4.02 (0.91)	.91	
	C2	3.68 (1.01)	.94	
	C3	4.05 (0.88)	.90	
	C4	4.05 (0.94)	.91	
	C5	3.91 (1.01)	.90	

**Table 3:** Correlation analysis (Pearson *r* and two-tailed *P*) among the research variables.

Variable		Gender	Age	Highest education	Whether medical staff	Perceived ease of use	Perceived usefulness	Use willingness
**Gender**								
	*r*	1	−0.042	0.150	0.273^a^	−0.177	−0.004	−0.079
	*P* value	—^b^	.66	.11	.003	.06	.96	.40
**Age**								
	*r*	−0.042	1	−0.451^a^	−0.263^a^	−0.168	−0.034	−0.078
	*P* value	.66	—	<.001	.005	.08	.72	.41
**Highest education**									
	*r*	0.150	−0.451^a^	1	0.395^a^	0.079	0.115	0.117
	*P* value	.11	<.001	—	<.001	.41	.23	.28
**Whether medical staff**								
	*r*	0.273^a^	−0.263^a^	0.395^a^	1	0.085	0.157	0.116
	*P* value	.003	.005	<.001	—	.37	.10	.22
**Perceived ease of use**									
	*r*	−0.177	−0.168	0.079	0.085	1	0.752^a^	0.764^a^
	*P* value	.06	.08	.41	0.37	—	<.001	<.001
**Perceived usefulness**									
	*r*	−0.004	−0.034	0.115	0.157	0.752^a^	1	0.889^a^
	*P* value	.96	.72	.23	.10	<.001	—	<.001
**Use willingness**								
	*r*	−0.079	−0.078	0.117	0.116	0.764^a^	0.889^a^	1
	*P* value	.40	.41	.22	0.22	<.001	<.001	—

^a^The correlation is significant at a significance level of .01 (two-tailed).

^b^Not applicable.

The differential validity of the three main facets of this study is based on the confidence interval in which the correlation coefficient between two facets is estimated. The findings are shown in Table 4. All confidence intervals did not contain 1, so the validity differed.

**Table 4:** Trust interval and discriminant validity of the correlation coefficients.

Parameter	Estimate	Lower	Upper	*P* value
Perceived ease of use → Perceived usefulness	0.72	0.52	0.84	.001
Perceived usefulness → Use willingness	0.93	0.86	0.97	.001
Perceived ease of use → Use willingness	0.20	−0.03	0.43	.05

**Figure 3:**
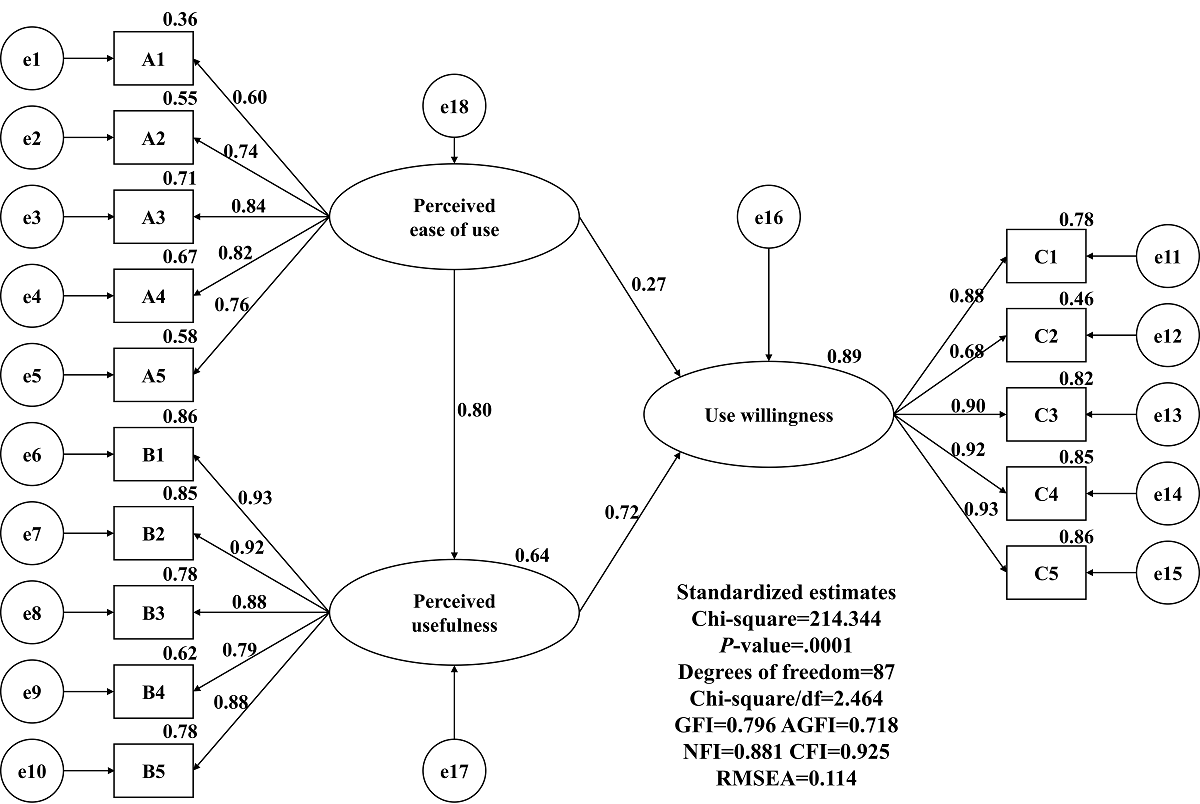
Standardized structural equations and hypothesis testing. GFI: goodness-of-fit index; AGFI: adjusted goodness-of-fit index; NFI: normed fit index; CFI: comparative fit index; RMSEA: root mean square error of approximation.

**Figure 4:**
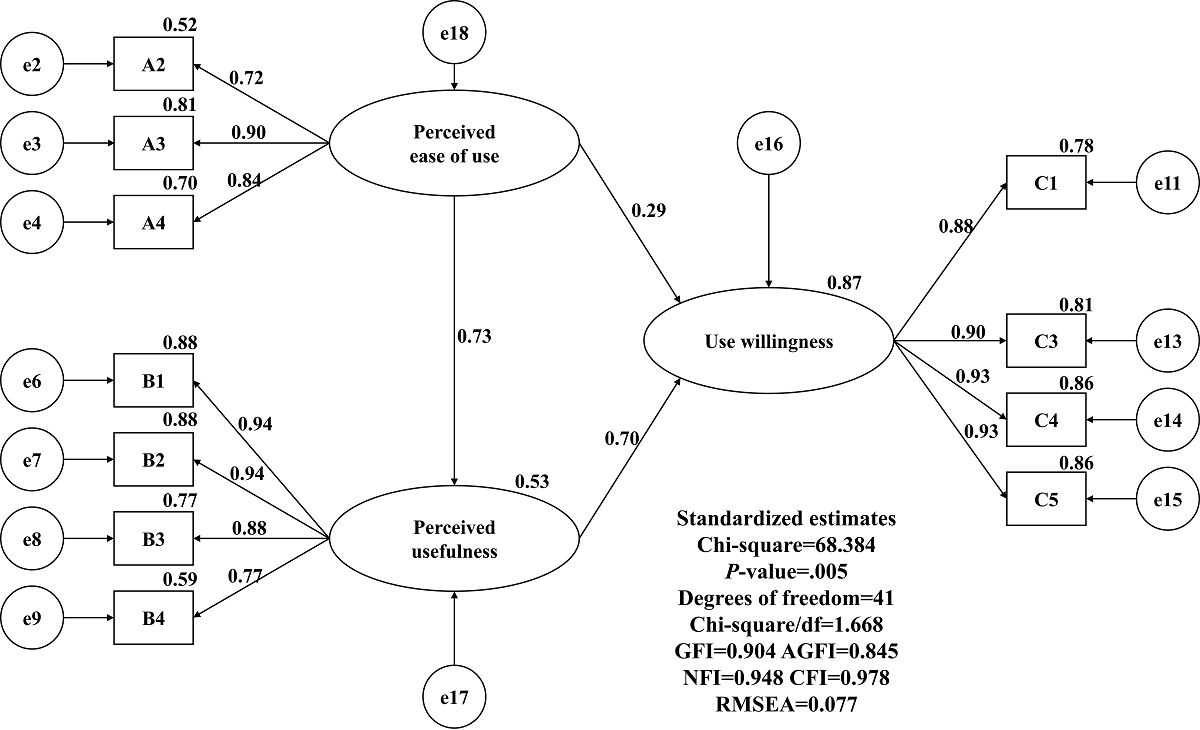
Modified normalized structural equation. GFI: goodness-of-fit index; AGFI: adjusted goodness-of-fit index; NFI: normed fit index; CFI: comparative fit index; RMSEA: root mean square error of approximation.

We also used a structural equation of verification. The verification results are shown in Figure 3. As the structural model adaptation degree in this study did not reach the general recommendation standard, the structural equations with collinearity doubts were deleted, as shown in Figure 4, and the adaptation test criteria were considered before and after the adjustment.

The standardized path coefficient reached the standard of significance, and perceived ease of use had a positive predictive power for perceived usefulness. The standardized path coefficient was 0.73, and the explanatory variation was 53%. Moreover, perceived ease of use had a positive predictive power for use willingness, with a standardized path coefficient of 0.29. Furthermore, perceived usefulness had a positive predictive power for use willingness, with a standardized path coefficient of 0.70 and an explanatory variation of 87%. The findings of this study show that perceived ease of use and perceived usefulness are significantly positively correlated (*P*=.001), perceived usefulness is significantly positively correlated with use willingness and frequency (*P*=.001), and perceived ease of use is significantly positively correlated with use willingness (*P*=.011). The standardized regression coefficients and significance are shown in Table 5.

**Table 5:** Standardized coefficients and significance of direct relationships.

Parameter	Estimate	Lower	Upper	*P* value
Perceived ease of use → Perceived usefulness	0.87	0.63	1.11	.001
Perceived ease of use → Use willingness	0.33	0.09	0.61	.001
Perceived usefulness → Use willingness	0.68	0.43	0.97	.001

Based on the results of the questionnaire survey, the study conducted an in-depth interview with six experts in three different fields, including law, medical, and technology fields. The three aspects of the development of an emergency and mutual-aid app model were discussed separately. The expert opinions at each level are presented below.

According to the survey results in this study, both perceived ease of use and perceived usefulness affect use willingness. If individuals want to implement an emergency and mutual-aid app model in Taiwan, they need to increase public willingness to use the app, as well as design a convenient user interface to improve perceived ease of use.

## Discussion

### Principal Findings

This study evaluated the feasibility of an emergency and mutual-aid app model in Taiwan and provided a reference for government policy. As the TAM helps in evaluation, users’ behavioral intention to use the app might be impacted by perceived usefulness and attitude toward the system [26,27]. The results indicated that perceived ease of use, perceived usefulness, and use willingness are important factors for users’ behavioral intention of using the app. Perceived usefulness had a strong relationship with use willingness. The availability and acceptability of an emergency and mutual-aid app model might make it an effective tool to assist people in managing emergencies and reducing mortality. The findings of this study indicate that the three TAM facets (perceived ease of use, perceived usefulness, and use willingness) support the findings of various studies about the impact of the three facets on the adoption of various forms of technology [28-31] and the tendency of people to use location-based emergency apps or any other health care apps [32-34].

### Public Health Implications

Perceived ease of use is an important factor influencing user acceptance and willingness to use information technologies, but it depends on a user’s experience with information technology [35]. Among the three facets, self-efficacy of an emergency and mutual-aid app (perceived ease of use) had the lowest score. However, “I could use an emergency and mutual-aid app even if no one tells me how to use it” had the lowest score among five questionnaires. Furthermore, “I could use an emergency and mutual-aid app if I have experience using other software similar to it” had an average score of 3.74. Some of the scores for these two questions were lower than the average score of the perceived ease of use facet, which may be because the respondents did not actually have any experience of using an emergency and mutual-aid app. Participants only relied on their experience of using other apps in the past and the description from the questionnaire. Several published studies mentioned that the effects of perceived usefulness and use willingness on technology adoption are higher than that of perceived ease of use [36,37]. When perceived ease of use and attitude toward information quality are positive, perceived usefulness is often high [38-40]. These findings might help in app design, and developers of an emergency app will need to provide a service that is easy to use, clear and understandable, easy to learn to operate, and easy to navigate and that has clear help messages [41-45].

The perceived usefulness of a location-based mobile app for emergency patient care is a crucial element behind an individual’s positive attitude toward using these types of apps and the behavioral intention regarding these apps in the future. User attitude for an emergency and mutual-aid app (perceived usefulness) had the highest score in our study. The services were perceived to be highly useful because (1) participants believed that the model is able to shorten the waiting time for ambulances and increase the chance of survival; (2) the model can function well and provide timely rescue to patients; (3) the probability that patients easily connect to various hospital websites and resources on different occasions is high; and (4) participants felt that the community first-aid resources could be better integrated and more effective. The results indicate that respondents maintained a positive attitude toward an emergency and mutual-aid app, which is supported by the findings in previous studies [46,47].

This study examined whether medical practitioners had a higher level of recognition of an emergency and mutual-aid app model than nonmedical practitioners. In particular, the basic information regarding occupation in the questionnaire assessed whether participants were medical staff. The three facets were perceived ease of use, perceived usefulness, and use willingness. Additionally, the three facets were not significant in terms of whether participants were medical staff (*P*=.37), and there were no significant differences among the facets discussed in this study (*P*=.22). With regard to age and the three facets in variance analysis, age and the three facets were not significant, and age was not significantly different for the facets discussed in this study. Gender, age, and whether medical staff were not significantly different for the three aspects of perceived ease of use, perceived usefulness, and use willingness in this study [48]. This study examined how the three research facets interact with each other according to structural equation verification. The findings show that perceived ease of use had a positive and significant influence on perceived usefulness (*P*<.001), perceived ease of use had a positive and significant influence on use willingness (*P*<.001), perceived usefulness had a positive and significant influence on use willingness (*P*<.001), and perceived usefulness had an intermediary influence on perceived ease of use and use willingness. Perceived ease of use had a positive predictive power for perceived usefulness, with a standardized path coefficient of 0.73 and an explained variation of 53%. Perceived ease of use had a positive predictive power for use willingness, with a standardized path coefficient of 0.29. Moreover, perceived usefulness had a positive predictive power for use willingness, with a standardized path coefficient of 0.70. Furthermore, perceived ease of use and perceived usefulness accounted for 87% of the interpretation of use willingness. Additionally, there was a complete effect of perceived ease of use on use willingness (coefficient of 0.93), and the confidence interval range was 0.69 to 1.27. It can be judged that perceived ease of use affects not only perceived usefulness but also use willingness.

According to the results of the survey, perceived ease of use and perceived usefulness affected use willingness, and experts, such as those from law, medical, and technical fields, developed approaches to enhance perceived ease of use and perceived usefulness for an emergency and mutual-aid app. They further enhanced use willingness and explored the corresponding policies for the feasibility and recommendation of an emergency and mutual-aid app [49,50]. Moreover, from in-depth interviews with experts, such as those from law, medical, and technical fields, it is known that if the heartbeat and blood flow stop and the organs are hypoxic, brain death might occur after 4 to 6 minutes, and it is important to avoid wasting the golden rescue time [51,52]. During this period, we assume that other people nearby can immediately perform CPR and use the AED to determine whether an electric shock is needed for a patient whose heart has stopped. The public has low willingness to perform CPR or use the AED for an unknown patient. It is recommended to pass a decree, use online media, or provide free first aid. Additionally, relevant courses or other methods can be used for publicity.

Science and technology experts believe that current technology can help develop an emergency and mutual-aid app, mainly depending on the development of labor resources and follow-up system maintenance [53-55]. The user interface should be considered in the use of the interface design to enhance perceived ease of use, and it is recommended to integrate different system resources. Emergency staff or ambulances can help avoid wasting the golden rescue time through information equipment and programming [56,57]. Experts mentioned that the main aspects of the success of emergency aid are implementation of CPR as soon as possible, collection of the electric shock device as soon as possible, and transport of the patient to a hospital as soon as possible. With an emergency and mutual-aid app, volunteer rescuers can immediately arrive at the accident location, and the information can be combined with a map of AEDs at public places. This might greatly reduce the waiting time of patients with heartbeat issues for CPR and AED use and might help gain the golden rescue time [58]. It is recommended that government agencies develop an emergency and mutual-aid app in Taiwan and enhance the willingness of people to use the app by implementing support policies and measures, publicizing basic public first-aid training, and promoting AEDs in public places to increase density. For a patient with heartbeat issues, information on a volunteer rescuer’s mobile phone, access to cardiopulmonary resuscitation, and use of electric shocks as soon as possible can help address the patient’s problem. This would ultimately improve the survival rate.

The main factors associated with the success of emergency aid are implementation of CPR as soon as possible, collection of the electric shock device as soon as possible, and transport of the patient to a hospital as soon as possible [59]. If an emergency and mutual-aid app is available, volunteer rescuers can immediately arrive at the accident location, and when combined with a map of AEDs at public places, it can greatly reduce the waiting time of patients with heartbeat issues for CPR and AED use, which might help gain the golden rescue time and improve the success rate of rescue [60]. The advent of the Internet of Things and big data will provide great help to mobile medical care [61,62]. According to the findings of the survey, we suggest that use willingness should be increased by enhancing perceived ease of use and perceived usefulness. It is necessary to take into account the user’s habits in the design of the app and pay attention to the operation of the interface [63,64]. This will help users easily start using the app. It is also recommended to connect different system resources in the future. For example, an emergency and mutual-aid app could directly connect to a control center, mobile ambulance, personal cloud medical information system, and medical hospital after reservation. The system might help ambulance staff to provide emergency services through valuable information, equipment, and programming. It therefore helps to avoid wasting the golden rescue time. This will allow the app to have the best benefits.

### Strengths and Limitations

The strength of our study is that it explored the use willingness, feasibility, and promotion strategies for the implementation of an emergency and mutual-aid app model in Taiwan through questionnaires and in-depth interviews with experts. However, our study has several limitations that need to be addressed. First, there is an issue with the research design, as the cost of developing and maintaining the app was not considered. For the development of an actual app, we need to consider the cost of research and development or the budget allocated by government agencies for research and development. Second, Taiwan does not have an actual emergency and mutual-aid app yet; therefore, in the questionnaire survey, respondents could not provide information about the actual experience of using such an app, which might have affected the results of the responses and statistical analysis. Third, regarding the questionnaire, in order to facilitate sampling, we did not use large-scale surveys, and this might have affected the findings of the statistical analysis. Finally, we conducted in-depth interviews with experts, and six experts were interviewed according to the purpose of this study. However, these experts were from different fields and had different experiences, and countermeasure suggestions might not cover all aspects that should be considered. Furthermore, each expert had an interview only once, and there was no meeting to facilitate the integration and exchange of ideas from experts in different fields. Indeed, this study discussed only preliminary ideas, and we look forward to having discussions with more experts and scholars when the development of an emergency and mutual-aid app is complete.

### Conclusion

The investigation of a mutual-aid mobile app regarding emergency care acceptance is relatively innovative for health care providers and policymakers. This study used the innovative aspects of perceived ease of use, perceived usefulness, use willingness, and behavioral use intention and proposed the TAM to evaluate users’ acceptance of a mutual-aid mobile app for emergency care. The findings of this study might help the government and policymakers to take decisions on the development of an emergency and mutual-aid app in Taiwan. The findings also suggest that the design should specifically consider increasing convenience and public recognition of the app. User approval of the app could effectively improve use willingness, which could achieve the goal of avoiding wastage of the golden rescue time. Furthermore, findings from our study show that experts believe the development of such an emergency and mutual-aid app model can be undertaken in Taiwan. Professional medical staff who identify patients with heartbeat issues should implement CPR as soon as possible and obtain the electric shock device as soon as possible. This will help to transport the patient to a hospital as soon as possible, reduce irreversible organ damage, and increase survival.

## Appendix

Multimedia Appendix 1 Research questionnaire.

## Abbreviations

AED: automated external defibrillator
CPR: cardiopulmonary resuscitation
TAM: technology acceptance model
