# Supplementary material for: Mutual-Aid Mobile App for Emergency Care: Feasibility Study
Source: JMIR Form Res. 2020 Mar 19;4(3):e15494. doi: 10.2196/15494 (PMC7118550; doi:10.2196/15494)
Supplement: Multimedia Appendix 1 [file formative_v4i3e15494_app1.docx]

**Appendix:**

Research questionnaire

Hello:

This is an academic use questionnaire, which is mainly about the feasibility assessment (using cognition and willingness) of the "emergency and mutual aid APP model" in Taiwan. Regardless of whether you have the useing experience of “emergency and mutual aid APP model”, please answers the following questions according to your own experience.

**The "emergency and mutual aid APP model" allows the user to choose whether he/she has been trained by professional first aid training, and is willing to assist strangers. If you select the consent, when receiving the first aid demand, it will send the patient location message through the satellite positioning system on the iPhone to the nearest emergency volunteer for rescue .**

The results of this study are for academic use only, and the information you provide is absolutely confidential, so please feel free to answer. Your support and cooperation are the key factors for the successful completion of this research. Thank you very much for taking the time to answer the questions

best regards

【Reply Instructions】：Please select the closest answer according to your current feeling level or situation. This is a multiple choice question, and please answer each question. Thank you for your cooperation.

I. Basic information: Please check or fill in your personal information as required by the project.

- 1. Gender：□male□female
  2. Your age：□20-40 years old □40-60 years old □60 years old or older □ other
  3. The highest degree you have completed：□junior high school or below □high school □bachelor degree □master degree or higher □other
  4. Your occupation：□student □military, public, and religious□service industry □financial industry□information technology □communication/advertising/design □art □free industry □medical industry □agriculture, forestry, animal husbandry □house management/ retirement □other

II. Self-efficacy of the emergency and mutual aid APP (perceived ease of use)

|  | Strongly disagree | disagree | Neutral | Agree | Strongly agree |
| --- | --- | --- | --- | --- | --- |
| A1. Even if no one around tells me how to use the "emergency and mutual aid APP", I can use it. |  |  |  |  |  |
| A2. I can use it as long as I have a manual for the "emergency and mutual aid APP". |  |  |  |  |  |
| A3. If I have plenty of time to let me learn how to use and operate the "emergency and mutual aid APP" and I can use it. |  |  |  |  |  |
| A4. If someone can let me request when I having problems with the use, I can use the " emergency and mutual aid APP" |  |  |  |  |  |
| A5. If I have used other software experiences similar to the "emergency and mutual aid APP", I can use it. |  |  |  |  |  |

III. Use attitude of the emergency and mutual aid APP (perceived usefulness)

|  | Strongly disagree | disagree | Neutral | Agree | Strongly agree |
| --- | --- | --- | --- | --- | --- |
| B1. I think that the "emergency and mutual aid APP model" is able to shorten the waiting time for ambulances and increase the chances of survival for those in need. |  |  |  |  |  |
| B2. I think that the "emergency and mutual aid APP model" can function well and provide timely rescue to patients. |  |  |  |  |  |
| B3. I think that when using the "emergency and mutual aid APP model", it is convenient for patients to easily connect to various hospital websites and resources in different occasions. |  |  |  |  |  |
| B4. If I need help from the "emergency and mutual aid APP model", I think I can get support quickly. |  |  |  |  |  |
| B5. On the whole, I feel that when using the "emergency and mutual aid APP model", the community's first aid resources can be better integrated and effective. |  |  |  |  |  |

IV. Willingness to use and frequency of the emergency and mutual aid APP

|  | Strongly disagree | disagree | Neutral | Agree | Strongly agree |
| --- | --- | --- | --- | --- | --- |
| C1. I am willing to provide the support needed for patients who need first aid through the "emergency and mutual aid APP model". |  |  |  |  |  |
| C2. I think it's easy to get first aid support through the "emergency and mutual aid APP model". |  |  |  |  |  |
| C3. In the future, if the environment permits, I am willing to use the "emergency and mutual aid APP model" as the resident program of the mobile phone. |  |  |  |  |  |
| C4. Overall, I feel that the "emergency and mutual aid APP model" is useful for the entire social environment. |  |  |  |  |  |
| C5. Overall, my willingness to use the "emergency and mutual aid APP model" is quite high. |  |  |  |  |  |
